# Malignant Transformation of Synovial Chondromatosis: A Systematic Review

**DOI:** 10.2174/1874325001711010517

**Published:** 2017-05-31

**Authors:** Vincent Y. Ng, Philip Louie, Stephanie Punt, Ernest U. Conrad

**Affiliations:** 1 Department of Orthopaedics, Greenebaum Cancer Center, University of Maryland Medical Center, 110 S. Paca St., 6^th^ Floor, Suite 300, Baltimore, MD 21201, USA; 2 Department of Orthopaedics, Rush University Medical Center, Chicago IL, USA; 3 Department of Orthopedics and Sports Medicine, Seattle Children’s Hospital, 4800 Sandpoint Way NE, Seattle, WA 98105, USA

**Keywords:** Synovial chondromatosis, Chondrosarcoma, Malignant transformation, Osteochondromatosis, Size

## Abstract

**Background:**

Synovial chondromatosis (SCh) can undergo malignant transformation. Pathologic diagnosis of secondary synovial chondrosarcoma (SChS) is challenging and misdiagnosis may result in over- or undertreatment.

**Method:**

A systematic review revealed 48 cases of SChS published in 27 reports since 1957. Data was collected to identify findings indicative of SChS and outcomes of treatment.

**Results:**

At median follow-up of 18 months, patients were reported as alive (10%), alive without disease (22%), alive with disease (15%), dead of disease (19%), dead of pulmonary embolism (4%), and unknown (29%). Initial diagnosis of SChS (grade: low/unknown 48%, intermediate/high 52%) was after biopsy in 58%, local resection in 29%, and amputation in 13%. Seventy-four percent of patients underwent 1.8 (mean) resections. Patients treated prior to 1992 were managed with amputation in 79% of cases compared to 48% after 1992. Symptoms were present for 72 mos prior to diagnosis of SChS.

Synovial chondrosarcoma demonstrated symptom progression over several months (82%), rapid recurrence after complete resection (30%), and medullary canal invasion (43%). The SChS tumor dimensions were seldom quantified.

**Conclusion:**

Malignant degeneration of synovial chondromatosis is rare but can necessitate morbid surgery or result in death. Pathognomonic signs for SChS including intramedullary infiltration are present in the minority of cases. Progression of symptoms, quick local recurrence, and muscle infiltration are more suggestive of SChS. Periarticular cortical erosion, extra-capsular extension, and metaplastic chondroid features are non-specific. Although poorly documented for SChS, tumor size is a strong indicator of malignancy.

Biopsy and partial resection are prone to diagnostic error. Surgical decisions are frequently based on size and clinical appearance and may be in conflict with pathologic diagnosis.

## INTRODUCTION

Synovial chondromatosis (SCh) is a metaplastic chondroid proliferation within articular joints and, less commonly, in bursa or tendon sheath [1-3]. The vast majority of cases are symptomatic yet benign [4]. While amenable to local resection, SCh is prone to recurrence (~15-25%), particularly if incompletely excised [4-7] Malignant transformation of SCh into secondary synovial chondrosarcoma (SChS) has been reported in 1-10% of patients [4, 8-10]. Because SCh often exhibits aggressive clinical, radiographic and histologic features, distinguishing SCh from SChS can be challenging [11, 12].

It is well recognized that most cartilaginous conditions such as SCh and multiple enchondromatosis represent low-grade or benign cartilage tumors on histologic analysis. Patients with SChS are often underdiagnosed and undergo multiple resections before SChS is considered as a diagnosis and adequate treatment is performed. The histologic challenges of diagnosing chondrosarcoma are well-recognized for low grade cartilage tumors [13].

Malignant transformation of SCh has previously been reported in the literature through numerous case reports and series. The purpose of this systematic review was to 1) identify clinical, radiographic and histopathologic findings indicative of SChS in the setting of SCh, 2) determine the outcomes of SChS, and 3) formulate a treatment approach for SChS.

## MATERIALS AND METHODS

A literature search on malignant transformation of SCh was performed for all studies indexed on PubMed and for cited references in aforementioned studies up to June 1^st^, 2014 (Table **1**). The search strategy consisted of terms synovial, chondromatosis, osteochondromatosis, malignant, chondrosarcoma combined with the Boolean operator AND Fig. (**1**). Case series of benign SCh were excluded. All cases of malignant transformation of SCh were included. Three non-English language articles were translated and reviewed as well. Forty-eight patients in 28 level IV evidence articles, as defined by Sackett [13], from 1957 to 2013 were identified [2, 4, 5, 7-9, 11, 12, 14-32] . No higher level of data were identified and the majority of reports consisted of less than 3 cases. Collected data included gender, age at diagnosis of SCh and SChS, size, osseous involvement on imaging (cortical *vs.* intramedullary), primary site of disease, presenting clinical symptoms, prior treatment for SCh, episodes of local recurrence, pathologic/histologic grade, clinical signs of transformation, method of SChS diagnosis, size of SChS lesion, treatment for SChS, metastatic spread, disease status and length of follow-up. Increasing pain over 6-12 weeks was interpreted as clinical progression. Early recurrence of SCh was defined as recurrence less than 12 months after resection.

An Excel worksheet (Microsoft, Redmond, WA) was created to compile and juxtapose all relevant data for comparison. The Fisher’s exact test was used to compare rates of amputation. Characteristics specific for neither SCh nor SChS were also noted (Table **1**).

## RESULTS

The mean age at diagnoses of SChS was 53 years. There were 52% males, 33% females, and 15% gender not reported. The sites of disease were knee (50%), hip (33%),, ankle (6%), shoulder (4%), and other/unknown (6%). At latest follow-up (median 18 mos; range, 3 to 136), outcomes were reported as alive (10%), alive without disease (22%), alive with disease (15%), dead of disease (19%), dead of pulmonary embolism (4%), and unknown (29%). Further specification regarding death from disease or pulmonary embolism was not available. The rate of local recurrence for SCh that underwent malignant transformation was very high. Seventy-four percent of patients underwent multiple resections with a mean of 1.8 resections (range, 1 to 3) and 61% eventually required amputation. Prior to 1992, the incidence of amputation was 79% (15 of 19) and after 1992, it was 48% (13 of 27) (p = 0.0017). Symptoms of pain and swelling were present for a mean of 72 months (3 to 462) prior to diagnosis of SChS. Initial diagnosis of SChS (grade: low 25%, intermediate 40%, high 12%, unknown 23%) was after biopsy in 58%, local resection in 29%, and amputation in 13%.

The following attributes were documented by the reporting authors in the following proportion of cases for SChS: rapid progression of pain (82%), invasion of medullary bone on imaging or histology (43%), rapid recurrence (<12 mos) after complete resection of SCh (30%), or infiltration of adjacent muscle (12%).

A soft tissue mass, joint effusion, joint space destruction, osseous erosion, local recurrence, and pathologic features of low-grade chondrosarcoma were universally reported as not specific for either SCh or SChS [4, 8, 18, 20, 23, 24, 26, 31]. The SChS dimensions were seldom quantified (range, 7-37 cm; 83% not reported), but the lesions were invariably larger than pre-existing SCh and had substantial involvement of surrounding tissues.

## DISCUSSIONS

**Fig. (2):**
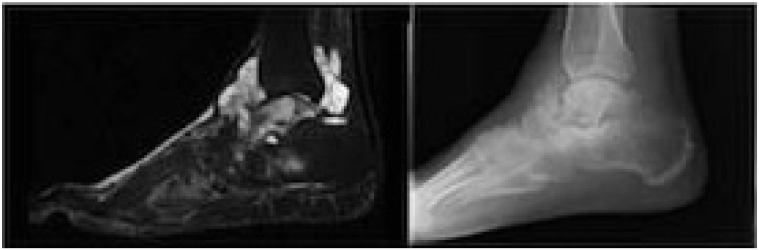
Synovial chondrosarcoma in the setting of synovial chondromatosis 47 year-old male with two prior ankle injuries and pain over the past 3 years now presenting with recent progression of pain and swelling. Initial biopsy was consistent with grade II/III chondrosarcoma, confirming the radiographic appearance (MRI, A; Plain XR, B) and clinical presentation. He underwent below-knee amputation.

**Fig. (3):**
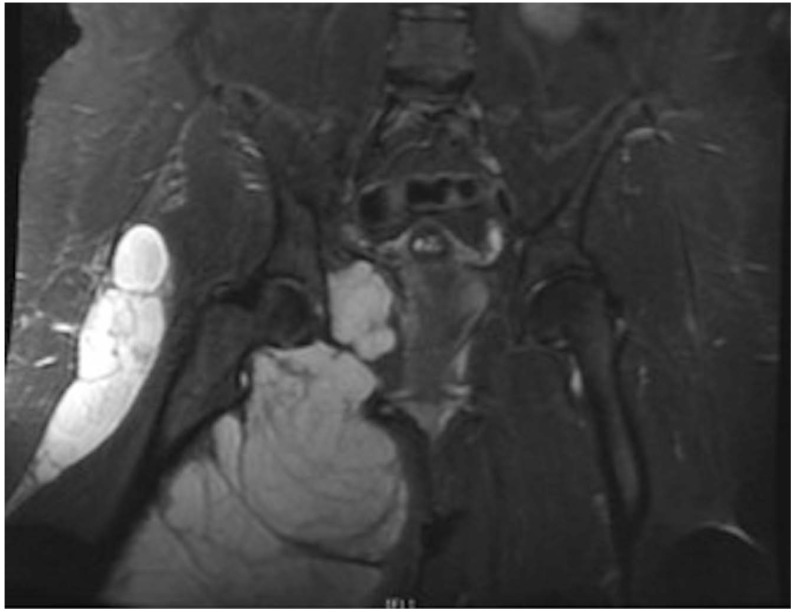
Synovial chondrosarcoma in the setting of synovial chondromatosis 43 year-old female presenting with a massive localized chondroid lesion suspected to have malignant degeneration of synovial chondromatosis based on imaging (MRI, A) and symptomatology. Histologic evidence of chondrosarcoma, however, was only detected after resection of essentially the entire lesion.

Differentiating SChS from benign SCh is a significant histologic and clinical challenge at presentation and following resection. Based on this systematic review of reported cases, objective diagnostic findings for SChS include medullary bone invasion on imaging or histology and pathologic findings consistent with grade II or III chondrosarcoma [30, 33, 34] (Fig. **2**). Concerning features include rapid clinical progression of pain, local recurrence within 12 mos after resection, soft tissue infiltration of tumor, and large size [34] (Fig. **3**). Similar to the diagnostic and grading challenges of other chondroid malignancies, accurate assessment of SChS requires consideration of clinical, radiographic and histologic findings [32].

Distinguishing benign from malignant chondroid tumors is notoriously difficult on pathology alone [35]. A benign or even low-grade pathologic diagnosis should be highly suspicious in the setting of a massive tumor. Size is a strong indicator of aggressive and malignant behavior. The size of SChS was frequently implied, but documentation of dimensions was absent in most reports. Although not well-recognized for SChS, size and location are well-described for assessing the risk profiles for conventional chondrosarcoma [36, 37] and other musculoskeletal malignancies [38, 39].

Benign cartilaginous conditions such as Ollier’s disease and SCh are recognized to have a more aggressive histologic appearance than intraosseous cartilage lesions or enchondromas [2, 26]. For SCh, findings such as periarticular bone or articular cartilage erosion, extra-capsular extension, and metaplastic chondroid features, even resembling low-grade chondrosarcoma, can be present. Because the relative invasiveness and metastatic potential of cartilage neoplasms lies along a continuum (Table **2**), making surgical decisions about the extent of resection can be difficult.

Limitations of this study include the availability of only Level IV evidence and multiple studies where tumor size is not quantified and long-term patient survival is not documented. Because of the rarity of this condition, extrapolations need to be made from the existing literature that may not be supported by large cohort or controlled studies. The treatment for SChS has evolved to include more limb preservation options rather than amputation, but because sample size is relatively limited and the variability between characteristics of each case, it is difficult to draw broad conclusions or recommendations regarding limb preservation.

The standard initial treatment for symptomatic SCh is joint-preserving arthroscopic or open resection [9, 29]. Incomplete removal of SCh and partial synovectomy will likely result in local recurrence [28]. Repeated local recurrence may be associated with eventual malignant transformation [7]. Seventy-five percent of SChS cases had multiple prior excisions of locally recurrent SCh.

 Depending on the extent of disease, total joint arthroplasty may be necessary. For patients with large tumors (>5-10 cm) and local invasion, open biopsy and incomplete resection are likely to result in a benign or low-grade cartilaginous neoplasm diagnosis [9, 10, 16, 17, 30]. For low-grade SChS, complete resection and close surveillance is probably adequate [33]. 

If there is local recurrence of a low-grade SChS or the presence of intermediate- or high-grade SChS on pathologic evaluation, wide resection is necessary and in many cases, amputation will be required to achieve adequate margins. Surgical decisions regarding large or recurrent tumors should be made on the basis of tumor size and imaging appearance, even with an apparent histologic diagnosis of “low-grade” chondrosarcoma.

## Figures and Tables

**Fig. (1):**
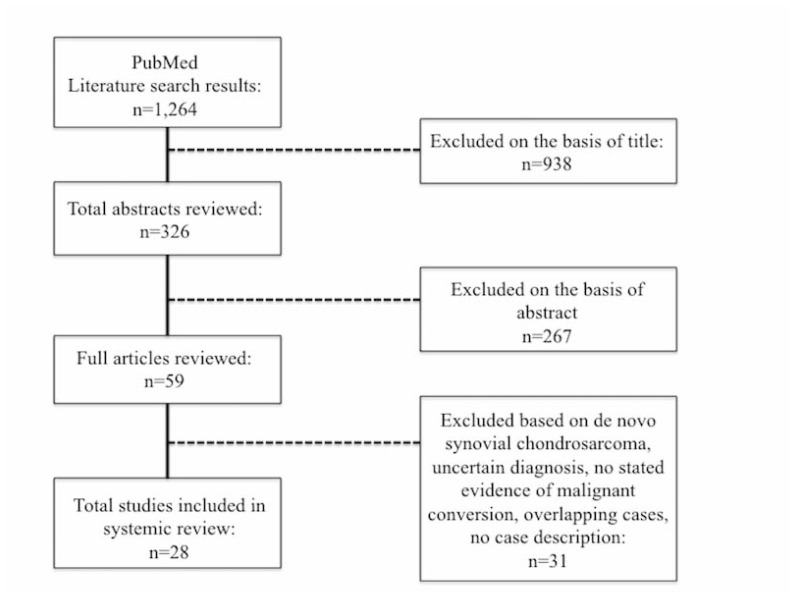
Search Flow Diagram.

**Table 1:** Description of studies included in systematic review.

**Authors**	**Year Published**	**No. of Cases**	**Surgical Treatment for SChS**	**Outcomes**
Brannon *et al*.	1957	1	Amp	Dead
Nixon *et al*.	1960	1	Amp	Unknown
Goldman *et al*.	1964	3	Amp (2), none (1)	Unknown (2), Alive unspecified (1)
Mullins *et al*.	1965	1	Amp	Alive w/ disease
King *et al*.	1967	1	Amp	Alive w/out disease
Dunn *et al*.	1974	1	Amp	Alive w/ disease
Milgram *et al*.	1976	1	None	Dead
Kaiser *et al*.	1980	1	Amp	Unknown
Hamilton *et al*.	1987	1	Amp	Alive unspecified
Garz *et al*.	1988	1	Resection / Megaprosthesis	Alive w/ disease
Perry *et al*.	1988	1	Amp	Dead
Benoit *et al*.	1990	1	Amp	Unknown
Bertoni *et al*.	1991	5	Amp (4), Synovectomy (1)	Alive w/out disease (4), Dead (1)
Kenan *et al*.	1993	1	Synovectomy	Alive with disease
Ontell *et al*.	1994	1	Amp	Alive without disease
Anract *et al*.	1996	6	Amp (4), Resection / Megaprosthesis (2)	Unknown (5), Alive without disease (1)
Hermann *et al*.	1997	1	None	Dead
Taconis *et al*.	1997	1	None	Dead
Wuisman *et al*.	1997	2	En-bloc resection (2)	Alive without disease (2)
Davis *et al*.	1998	2	Amp (1), None (1)	Dead (2)
Hallam *et al*.	2001	1	TKA	Alive without disease
Wittkop *et al*.	2002	2	Unk (2)	Unknown (2)
Sperling *et al*.	2003	2	Amp (1), None (1)	Alive with disease (2)
Bhadra *et al*.	2007	1	Amp	Alive unspecified
Sah *et al*.	2007	1	Amp	Unknown
Campanacci *et al*.	2008	2	Amputation (1), Palliative Radiation (1)	Alive without disease (1), Alive with disease (1)
Yao *et al*.	2012	1	TKA	Unknown
Evans *et al*.	2013	5	Amp (3), Debulking (2)	Alive unspecified (2), Dead (3)

Legend – Amp (amputation); DOD (dead of disease); Unk (unknown/not reported); AWD (alive with disease); NED (alive; no evidence of disease); TKA (total knee arthroplasty).

**Table 2:** Spectrum of local invasiveness and metastatic potential for cartilaginous neoplasms.

**Chondroma / Enchondroma**	**Increasing Invasiveness**
**Multiple Enchondromatosis**	**Metastatic Potential**
**Synovial Chondromatosis**	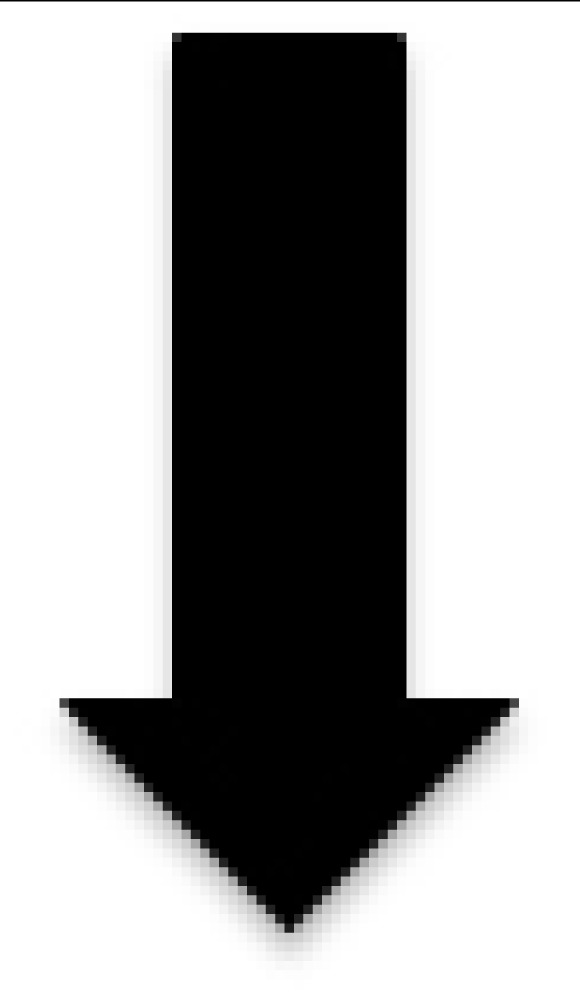
**Chondroblastoma**	
**Low Grade Chondrosarcoma**	
**Intermediate Grade Chondrosarcoma**	
**High Grade Chodrosarcoma**	
**Dedifferentiated Chondrosarcoma**	
